# Biochemical and molecular characterization of 3-Methylcrotonylglycinuria in an Italian asymptomatic girl

**DOI:** 10.1590/1678-4685-GMB-2017-0093

**Published:** 2018-05-14

**Authors:** Carla Cozzolino, Guglielmo RD Villani, Giulia Frisso, Emanuela Scolamiero, Lucia Albano, Giovanna Gallo, Roberta Romanelli, Margherita Ruoppolo

**Affiliations:** 1 CEINGE Biotecnologie Avanzate CEINGE Biotecnologie Avanzate Naples Italy CEINGE Biotecnologie Avanzate, Naples, Italy; 2 Università degli Studi di Napoli Università degli Studi di Napoli Dipartimento di Medicina Molecolare e Biotecnologie Mediche Naples Italy Dipartimento di Medicina Molecolare e Biotecnologie Mediche, Università degli Studi di Napoli, “Federico II”, Naples, Italy

**Keywords:** 3-Methylcrotonylglycinuria, MCCC2 mutations, 3-methylcrotonyl-CoA carboxylase deficiency, newborn screening, organic aciduria

## Abstract

3-Methylcrotonylglycinuria is an organic aciduria resulting from deficiency of 3-methylcrotonyl-CoA carboxylase (3-MCC), a biotin-dependent mitochondrial enzym carboxylating 3-methylcrotonyl-CoA to 3-methylglutaconyl-CoA during leucine catabolism. Its deficiency, due to mutations on *MCCC1* and *MCCC2* genes, leads to accumulation of 3-methylcrotonyl-CoA metabolites in blood and/or urine, primarily 3-hydroxyisovaleryl-carnitine (C5-OH) in plasma and 3-methylcrotonyl-glycine (3-MCG) and 3-hydroxyisovaleric acid (3-HIVA) in the urine. The phenotype of 3-MCC deficiency is highly variable, ranging from severe neurological abnormalities and death in infancy to asymptomatic adults. Here we report the biochemical and molecular characterization of an Italian asymptomatic girl, positive for the newborn screening test. Molecular analysis showed two mutations in the *MCCC2* gene, an already described missense mutation, c.691A > T (p.I231F), and a novel splicing mutation, c.1150-1G > A. We characterized the expression profile of the splice mutation by functional studies.

## Introduction

**Figure 1:**
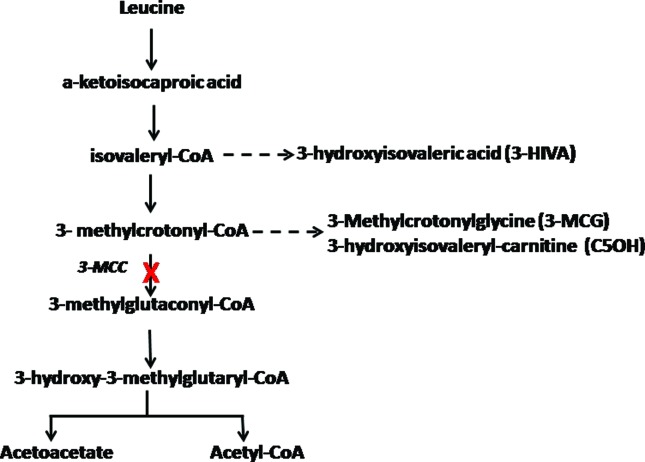
The 3-MCC-catalyzed reaction and its position in the leucine catabolic pathway. The dashed arrow indicates the metabolites that accumulate due to deficiency of 3-MCC.

3-Methylcrotonylglycinuria (OMIM #210200, #210210) is an organic aciduria resulting from deficiency of 3-methylcrotonyl-CoA carboxylase (3-MCC, EC 6.4.1.4), a biotin-dependent mitochondrial enzyme involved in the fourth step of the catabolic pathway of the amino acid leucine (Sweetman and Williams, 2001) (Figure 1). Its deficiency leads to accumulation in blood and/or urine of 3-methylcrotonyl-CoA metabolites, primarily 3-hydroxyisovaleryl-carnitine (C5-OH) in plasma and 3-methylcrotonyl-glycine (3-MCG) and 3-hydroxyisovaleric acid (3-HIVA) in urine (Arnold *et al.*, 2012); patients often show a secondary carnitine deficiency due to its conjugation to 3-HIVA. The 3-methylcrotonylglycinuria is the most frequent organic aciduria detected by an increase of C5-OH in tandem mass spectrometry-based newborn screening programs (NBS) (Gallardo *et al.*, 2001). Its prevalence is highly variable ranging from 1:2,400 in the Faroe Islands (Thomsen *et al.*, 2015) to 1:68,333 in the Chinese population (Yang *et al.*, 2015). Interestingly, the prevalence could be lower in Brazil, and in fact, studies on frequencies of primary disorders of organic acid metabolism in high-risk Brazilian patients evidenced a frequency of only 0.92% of 3-Methyl-crotonylglycine-CoA carboxylase deficiency (two patients among 218 with organic aciduria) (Wajner *et al.*, 2002; Wajner *et al.*, 2009).

3-MCC deficiency is inherited as an autosomal recessive trait due to mutations affecting one of the two genes, *MCCC1* and *MCCC2,* encoding for two subunits, MCCα and MCCβ, respectively, that form the 3-MCC protein (Baumgartner *et al.*, 2001). The α subunit includes the biotin carboxylase (BC) domain and the biotin carboxyl carrier protein domain covalently bound with a biotin prosthetic group, while the β subunit contains the carboxyltransferase (CT) domain (Grünert *et al.*, 2012).

The clinical phenotype of 3-MCC deficiency is highly variable, ranging from severe neurological abnormalities and death in infancy (Baykal *et al.*, 2005) to asymptomatic adults (Dantas *et al.*, 2005; Forsyth *et al.*, 2016). A severe presentation of 3-MCC deficit may include the Reye-like illness, ketoacidosis, hypoglycemia, hyperammonemia, psychomotor retardation, seizures, symptoms of cardiorespiratory failure and coma. Mild phenotypes often include fatigue and weakness during catabolic episodes or mild developmental delay. Moreover, cardiomyopathy, brain atrophy and fatty infiltration of liver or muscle may also occur (Grünert *et al.*, 2012). Nonetheless, most of the patients diagnosed by NBS as affected by 3-MCC deficiency appear to be asymptomatic during the whole life (Forsyth *et al.*, 2016).

In this report we present the biochemical and molecular characterization of an Italian girl, positive for the newborn screening test. Molecular analysis showed two mutations in *MCCC2* gene, a missense mutation and a splicing mutation. We characterized the expression profile of the novel splice mutation by functional studies.

## Materials and Methods

### Biochemical analysis

The biochemical characterization of the patient was obtained by analyzing amino acids and acylcarnitines in dried blood spot (DBS) (at three days from birth) and in serum (at seven days from birth), organic acids in urine and biotinidase enzyme activity on dried blood spot. For these analyses, labeled standards of amino acids and acylcarnitines were purchased from Cambridge Isotope Laboratories (Andover, MA, USA). External standard blood spots from acylcarnitines and amino acids were from CDC (Atlanta, GA, USA).

Amino acids and acylcarnitines were analyzed as butyl esters by using a triple quadrupole tandem mass spectrometer API 4000 (AB/Sciex) connected to an Agilent 1200 Series autosampler as described (Scolamiero *et al.*, 2014; Scolamiero *et al.*, 2015). Briefly, DBS from the newborn was punched into a 1.5 mL test tube and 200 μL of methanol containing labeled standards were added.

The standard concentrations were in the 500–2500 μmol L^-1^ range for amino acids, and in the 7.6–152 μmol L^-1^ range for acylcarnitines. After 20 min of shaking at room temperature, the samples were dried under a nitrogen flow. The extracted acylcarnitines and amino acids were derivatized to butyl esters with 80 μL of 3 N HCl in N-butanol at 65 °C for 25 min. After derivatization, the samples were dried under nitrogen flow and resuspended in 300 μL of acetonitrile/water (70:30) containing 0.1% formic acid. Forty microliters were injected in the flow injection analysis mode for the MS/MS analysis. For serum analysis, 10 μL of the patient’s serum was spotted on a Schleicher &Schuell 903 grade filter paper (Whatman, Dassel, Germany) and dried overnight at room temperature. The next day, the filter paper was punched into a 1.5 mL test tube and processed as described for DBS. Each sample was analyzed on an API 4000 triple quadrupole mass spectrometer (Applied Biosystems-Sciex, Toronto, Canada) coupled with the high performance liquid chromatograph Agilent 1100 series (Agilent Technologies, Waldbronn, Germany), as described (Scolamiero *et al.*, 2015).

The extraction and quantification of the urinary organic acids was performed as previously reported (Villani *et al.*, 2016). Urine samples were quantified for creatinine in order to determine the amount of sample to be analyzed, equivalent to 0.5 μmol of creatinine, and NaOH 30% was added to the samples up to a final pH of 14.0. Subsequently, 500 μL of hydroxylamine hydrochloride 2.5 g/L (in water) were added to each tube and samples were allowed to react at 60 °C for 1 h. After acidification with H_2_SO_4_ 2.5 N to a final pH of 1.0 internal standards were added: 10, 20 or 20 μl of a 100 μg/mL solution of dimethylmalonic acid, tropic acid and pentadecanoic acid (PDA), respectively. Three extractions of the organic acids were performed by mixing vigorously each sample with 2 mL of ethyl acetate; the organic phases were transferred to another tube, and approximately 1 g of Na_2_SO_4_ was added. After 1 hour on bench, samples were centrifuged and the organic phase was transferred to a new clean glass tube to be completely evaporated under a gentle nitrogen flow. Fifty microliters of BSTFA was added to each dried sample and the derivatization reaction was performed at 60 °C for 30 min. Finally, 1 μL of the sample was used for GC/MS injection. Analyses were performed on an Agilent Technologies Model 7890A gas chromatograph combined with a 5975C mass spectrometer system and equipped with a split-mode capillary injection port held at 280 °C with a split ratio of 10:1. The column (Agilent J&W GC column HP-5MS) was directly interfaced to the ion source. The oven temperature was programmed from 70 °C to 280 °C at a rate of 10 °C/min, and the helium flow program was 1 mL/min. Data were acquired by repetitive scanning over a range of 50–550 amu. The retention time and area of each peak was automatically determined and printed out by the MSD Productivity Chemstation software (Agilent Technologies). Quantitation of each analyte was performed referring to the injected PDA quantity according to Tanaka *et al.* (1980a, b). The concentration of organic acids was normalized to the creatinine concentration of the urine sample and expressed as mmol organic acid/mol creatinine.

Neonatal screening for biotinidase deficiency was performed by colorimetric semi-quantitative analysis (Heard *et al.*, 1984).

### Genetic analysis

Genomic DNA was extracted from peripheral blood leukocytes of the patient and her parents using the “Nucleon” procedure (GE Healthcare, Little Chalfont, UK). Molecular analysis of the *MCCC1* (methylcrotonoyl-CoA carboxylase 1, alpha) and *MCCC2* (methylcrotonoyl-CoA carboxylase 2, beta) genes was performed by PCR, followed by direct sequencing of all exons and exon-intron boundaries, including 5’- and 3’-UTR regions (primers, sequences and condition for PCR amplification are available on request). PCR products were examined for sequence variations using a Big Dye Primer Cycle Sequencing kit and an ABI 3730 DNA Analyzer (Applied Biosystems). Nucleotide positions were numbered on the basis of the cDNA sequence (GenBank, *MCCC1: NM_020166.4* and *MCCC2: NM_022132.4*) according to the nomenclature of den Dunnen and Antonarakis (2001).

Since the patient’s RNA was not available for studying the effect of the c.1150-1G > A novel variation on splicing pattern, we used an artificial minigene construct designated pMGene (Amato *et al.*, 2012) kindly donated by F. Amato. A DNA fragment of approximately 1 kb, including the c.1150-1G > A variation in the *MCCC2* gene, was amplified from the genomic DNA of the patient and cloned into the pMGene: the PCR product was obtained using the Expand High Fidelity PCR System (Roche Life Science, Penzberg, Germany), then digested by the *Kpn*I restriction enzyme and cloned into the *Kpn*I-digested dephosphorylated pMGene vector (primers, sequences and conditions for PCR amplification and cloning are available on request). All clones were sequenced, and a wild-type and a mutated form were retained for expression experiments.

tsA201 cells were grown in DMEM supplemented with 10% fetal bovine serum and 1% penicillin/streptomycin in a humidified, 5% CO_2_ atmosphere at 37 °C. They were transfected with 2 μg of WT-pMGene or 2 μg of MUT-pMGene plasmids, using FuGENE 6 (Roche), according to the manufacturer’s instructions. Forty-eight hours after transfection, cells were collected and total RNA was extracted using TriPure Isolation Reagent (Roche Applied Science). Next, 1 μg of total RNA was reverse transcribed using SuperScript VILO cDNA synthesis kit (Life Technologies). Finally, 200 ng of cDNA were amplified using the forward primer pMG-GFP-FW (5’-ACGACGGCAACTACAAGACC-3’) and reverse primer pMG-bglob-ex3-REV (5’-CACACCAGCCACC ACTTTC-3’) in a previously described protocol (Catanzano *et al.*, 2010). The PCR products were subjected to bidirectional cycle sequencing using a Big Dye Primer Cycle Sequencing kit and an ABI 3730 DNA Analyzer (Applied Biosystems).

To verify the potential role of the variants identified by genetic screening, we also checked their frequency in population genomes databases, such as 1.000 Genomes (http://www.internationalgenome.org/) and ExAC (http://exac.broadinstitute.org/) consortia, which aggregate and harmonize exome sequencing data from a variety of large-scale sequencing projects. *In silico* bio-informatic evaluations were performed using Alamut Focus version 0.9, a licensed software package available from Interactive Biosoftware (www.interactive-biosoftware.com). Genomic sequences (WT and mutant) were processed by this predictor software using five splicing (SpliceSiteFinder-like, MaxEntScan, Neural Network Splice, GeneSplicer, and Human SplicingFinder) and three missense prediction algorithms (SIFT, Polyphen-2, MutationTaster). The robustness of these bio-informatic tools is widely accepted (Frisso *et al.*, 2016).

## Results

The patient was a girl born to non-consanguineous parents after a full term normal delivery of dizygotic twins, weighing \ 2.57 kg. Physical examination was normal and she was discharged from hospital 72 hours after birth. However, while the amino acid profile was normal (data not shown), the acylcarnitine profile, obtained from dried blood spot during the newborn screening, presented a significant increase in 3-hydroxyisovaleryl-carnitine (C5-OH), along with a normal free carnitine (C0) concentration (Table 1). Similarly, a high concentration of C5-OH with normal C0 concentration was reported as a consequence of liver immaturity in a false-positive case, having a blood C5-OH value normalized at five months of life (Rangel-Córdova *et al.*, 2009). In addition, a recent study pointed out that the C5-OH level found in newborn screening is not sufficient for diagnostic or predictive purposes (Forsyth *et al.*, 2016). However, the detection of a C5-OH concentration well above our cut-off led us to deepen the clinical evaluation and to start prompt confirmatory testing in order to investigate whether an inborn error of metabolism was associated to the biochemical alteration. The increase of C5-OH acylcarnitine in blood/plasma may be ascribed to different pathologies, since several enzymes (3-methylcrotonyl-CoA carboxylase, 3-methylglutaconic hydratase, 3-hydroxy-3-methylglutaryl-CoA lyase, 2-methyl-3-hydroxybutyril-CoA dehydrogenase, beta-ketothiolase, holocarboxylase synthetase and biotinidase) may be defective either in the leucine or the isoleucine catabolic pathway (Sweetman and Williams, 2001).

**Table 1:** Biomarkers found altered in the analyzed patient. These were quantified by LCMSMS analysis on dried blood spot and serum, by GCMS analysis in urine

	Dried Blood Spot (DBS) (μmol/L)	Serum (μmoli/L)	Urine (mmol/mol crea)
C5OH	4.09 (< 0.4)	4.58 (< 0.13)	-
C0	15.29 (11-51)	8.7 (10-45)	-
3-hydroxy-isovaleric acid	-	-	70 (0-1.3)
3-methylcrotonylglycine	-	-	441 (n.d.)

reference intervals are reported in brackets n.d.: not detectable.

**Figure 2:**
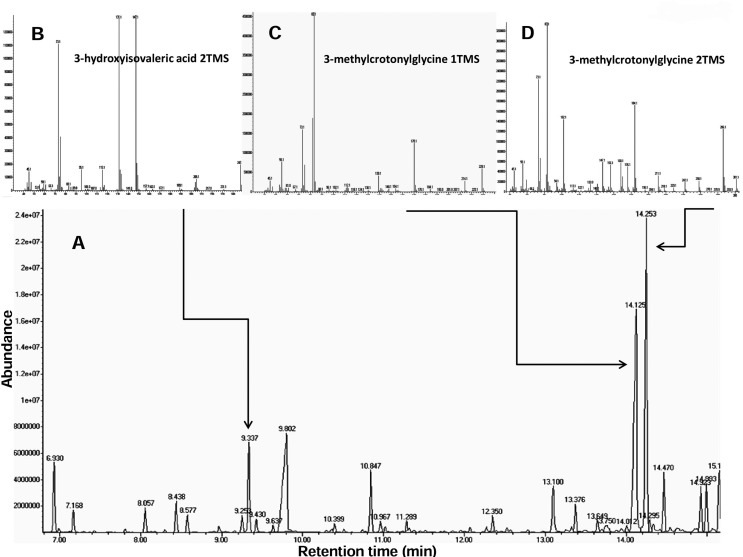
Patient’s urinary organic acids profile. (A) Partial organic acids profile from urine; arrows point to the main altered metabolites; (B-D) mass spectra for these organic acids. 1TMS and 2TMS refer to the presence of 1 or 2 trimethylsylil ester groups, respectively, added during the derivatization reaction.

Although the child remained asymptomatic, the acylcarnitine analysis performed on serum at 7 days from birth confirmed the increase of C5-OH, but showed also a significant decrease of C0 (Table 1). As stated above, elevation of C5-OH acylcarnitine levels in blood spot is not unique to 3-MCC deficiency. Conditions such as 3-hydroxy-3-methylglutaryl-coenzyme A lyase deficiency, β-ketothiolase deficiency, multiple carboxylase deficiency resulting from holocarboxylase synthetase or biotinidase deficiency, 2-methyl 3-hydroxybutyric acidemia, and 3-methylglutaconic aciduria may occur with elevation of C5OH acylcarnitine in asymptomatic infants. Therefore, the differential diagnosis between different organic acidurias must be considered in the presence of isolated elevation of C5OH acylcarnitine levels. In our case, the analysis of the newborn urinary organic acids showed an abnormal profile characterized by an increase of 3-hydroxyisovaleric acid and a massive excretion of 3-methyl-crotonylglycine (Table 1, Figure 2). A biotinidase enzyme assay on dried blood spot showed normal enzyme activity. A possible maternal origin of the metabolic alteration, due to transplacental transfer of C5OH (Rangel-Córdova *et al.*, 2009), was investigated and excluded by the analysis of the mother’s serum acylcarnitine and urine organic acids (data not shown). These results were consistent with the diagnosis of 3-methyl-crotonyl-CoA carboxylase deficiency and justified the molecular diagnosis.

**Figure 3:**
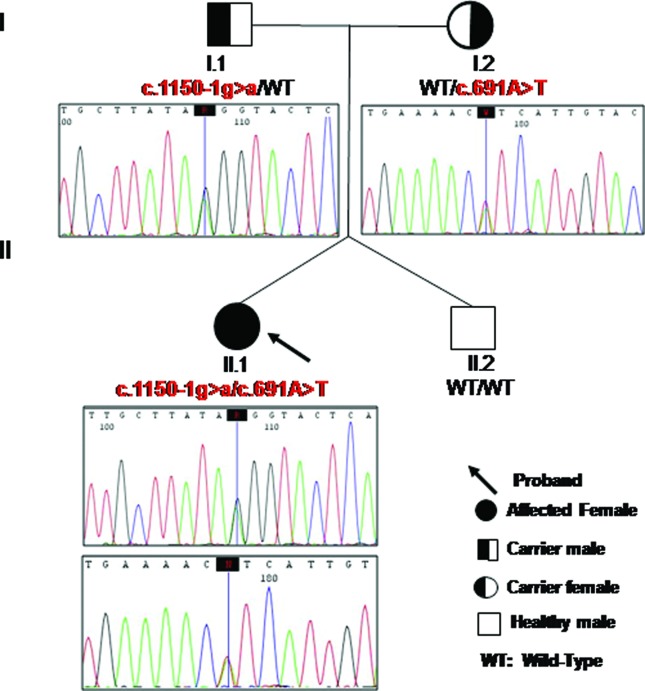
Pedigree of the patient with MCC deficiency. Sequence electropherograms of exon 7 and intron 12/exon 13 boundary of the *MCCC2* gene are shown for the patient and her parents.

3-MCC deficiency is due to mutations in either the *MCCC1* or *MCCC2 gene.* The Human Gene Mutation Database (HMGD) (Stenson *et al.*, 2014) currently includes 103 *MCCC1* mutations and 113 *MCCC2* mutations, most of which are missense mutations. Several of these mutations were recently reported in the Portuguese newborn screening program (Fonseca *et al.*, 2016). In our patient, the sequencing of the coding regions of *MCCC1* and *MCCC2* genes showed that she was a compound heterozygote for two mutations in the *MCCC2* gene: a missense mutation in exon 7, namely c.691A > T (p.I231F), and a possible splicing mutation in intron 12, c.1150-1G > A transition, inherited from the patient’s mother and father, respectively. Her twin brother did not carry these mutations (Figure 3). The c.691A > T (p.I231F) mutation was annotated in the HGMD database (http://www.hgmd.cf.ac.uk/), and showed an MAF = 0.02% and 0.0008% in the 1000 Genomes and ExAC databases, respectively. The c.1150-1G > A variant was absent from all the examined databases (HGMD, 1000 Genomes and ExAC).

The c.691A > T mutation causes the replacement of isoleucine 231, a non-polar amino acid, with phenylalanine, an aromatic amino acid (p.I231F). This variation has been described for the first time, in homozygosis, in an asymptomatic Iranian patient (Al-Jasmi *et al.*, 2016), positive in newborn screening (high levels of C5-OH). I231 is a highly conserved amino acid, and a deleterious effect is predicted for I231F-mutated protein by *in silico* analysis performed using Alamut software (Supplemental Table S1).

**Figure 4:**
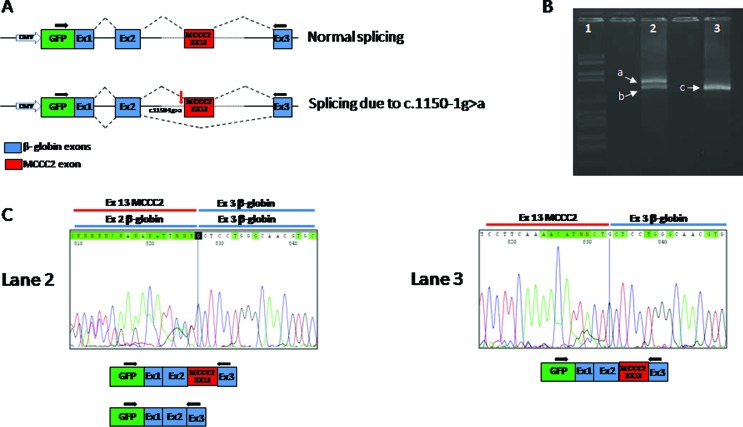
Minigene construct and RT-PCR results obtained in expression studies. (A) The minigene construct (pMGene) used in the study: a DNA fragment of approximately 1 kb was directly amplified from the genomic DNA of the patient and cloned into the pMGene as decribed in the Results section. All clones were sequenced, and a wild-type and a mutated clone were used for expression experiments. (B and C) The splicing pattern, evaluated by RT-PCR and sequence analysis of mRNA extracted from cell lines transfected with WT or mutated minigene constructs. Lane 1: molecular weight marker, lane 2: RT-PCR of RNA obtained using the mutant clone; lane 3: RT-PCR of RNA obtained from the normal clone.

The novel variant c.1150-1G > A is located in the acceptor splice site of intron 12 of the *MCCC2* gene and presumably resulted in the skipping of exon 13, as predicted by the bioinformatics program (Table S1). To verify this prediction, we used the minigene system, since the patient’s RNA from leucocytes for transcript analysis was unavailable. The RT-PCR and sequence analysis of mRNA, extracted from cell lines transfected with WT or mutated minigene constructs, revealed the presence of two transcripts (normal and alternative) in the pMGene-MCCC2-c.1150-1G>A, and only one (normal) in the pMGene-MCCC2-WT (Figure 4). The alternative transcript showed the skipping of exon 13, resulting in a frame shift starting at codon Gly384, and a stop codon 32 positions downstream (p.G384Dfs*32). The presence of these two mutations in the gene *MCCC2* definitively confirmed the diagnosis of 3-MCC deficiency for the patient.

## Discussion

Deficiency of 3-methylcrotonyl-CoA carboxylase does not show a good genotype-phenotype correlation, since deleterious mutations in homozygosis do not cause a clinical phenotype, and even complete absence of 3-MCC activity leads to clinical manifestations in a rather small subgroup of individuals (Dantas *et al.*, 2005; [Bibr B16] Morscher *et al.*, 2012).

In our study, an asymptomatic girl showed a significant alteration in biochemical markers. A persistent increase of C5-OH levels was detected by DBS and serum analysis, and a small decrease of C0 levels was demonstrated by serum acylcarnitine analysis at seven days from birth. An increase in 3-hydroxyisovaleric acid and a massive excretion of 3-methyl-crotonylglicine resulted from urinary organic acid quantization. The decrease, in serum, of C0 levels after four days from the first determination on DBS appears to be consistent with the persistent high levels of 3OH-isovaleryl-carnitine (C5-OH), which may gradually deplete the reserves of free carnitine.

Molecular analysis confirmed the diagnosis of 3-MCC deficiency. Interestingly, the patient presented two *MCCC2* mutations: the splicing mutation generating a frameshift most likely produces a null allele; the missense mutation (p.I231F) is located in the CT domain. It is difficult to establish the pathogenic role of a missense mutation; however, the deleterious effect predicted for the I231F-mutated protein by *in silico* analysis suggests that it may impair the carboxyltransferase activity.

Although the clinical relevance of the biochemical alterations related to this pathology is yet under evaluation, it is unquestionable that, in some patients, the 3-MCC defect results in severe clinical manifestations, mainly neurological impairment and cardiorespiratory failure, which can lead to death in infancy (Bannwart *et al.*, 1992; Dantas *et al.*, 2005), or acquires relevance later in the patient’s life. In these cases it is hypothesized that the 3-MCC enzyme defect cooperates with environmental factors or modifying genes to establish the pathological evolution of the disease ([Bibr B30] Baumgartner, 2005). Moreover, recent studies suggest that alterations of the cellular redox homeostasis may potentially be involved in the pathophysiology of 3-MCC deficiency (Zanatta *et al.*, 2013). Therefore, the accumulation of oxidative damage during the patient’s life could contribute to explain neurological symptoms in late onset 3-MCC forms, with or without previous crises of metabolic decompensation.

With this in mind, aim of the present report is, first of all, to emphasize the importance of differential diagnosis among much more severe metabolic diseases, in asymptomatic infants with high levels of C5OH. Moreover, a constant monitoring is recommended for patients presenting with 3-methylcrotonyl-CoA carboxylase deficiency, particularly asymptomatic newborns. The possible absence of symptoms should not lead parents and paediatrics to underestimate the pathology. It is also suggested, despite the apparent absence of clinical manifestations, to evaluate the opportunity of starting a preventive therapy in patients identified by abnormal biochemical findings, in order to reduce the risk of neurological delay and cardiomyopathy.

## Supplementary material

The following online material is available for this article:

Table S1 - Summary of bioinformatics analysis using the Alamut software.
