# Supplementary material for: Biochemical and molecular characterization of 3-Methylcrotonylglycinuria in an Italian asymptomatic girl
Source: Genet Mol Biol. 2018 May 14;41(2):379–85. doi: 10.1590/1678-4685-GMB-2017-0093 (PMC6082241; doi:10.1590/1678-4685-GMB-2017-0093)
Supplement: Supplementary file 1 [file 1415-4757-GMB-10159016784685GMB20170093-s001.pdf]

Supplementary Material to "Biochemical and molecular characterization of 3-Methylcrotonylglycinuria in an Italian asymptomatic girl"

Table S1 - Summary of bioinformatics analysis using the Alamut software. Square brackets show the score range for each algorithm, when applicable.

**Nucleotide variation cDNA**	**Aminoacid variation protein**	**POLYPHEN-2 [0;1]§**	**SIFT [0;1]***	**Mutation Taster**	**Splice Site Finder (0–100)**		**Max Ent Scan (0–16)**		**NNSPLICE (0–1)**		**Gene Splicer (0–15)**		**Human Splicing Finder (0–100)**		**Alamut Predicted Change**
					**WT**	**MUT**	**WT**	**MUT**	**WT**	**MUT**	**WT**	**MUT**	**WT**	**MUT**	
c.691A>T	p.I231F	0.986	0.01	Disease causing	NA	NA	NA	NA	NA	NA	NA	NA	NA	NA	Variant of uncertain significance
c.1150-1G>A	NA	NA	NA	NA	91.37	—	7.91	—	0.99	—	5.11	—	84.62	—	Acceptor splice site: -100%

§: the higher the value, the higher the probability of pathogenicity; *: probability of observing the new amino acid at that position, a value of between 0 and 0.05 is predicted to affect protein function. NA: Not Applicable; —: splice site not detected.
